# Laparoscopic Major Gastrointestinal Surgery Is Safe for Properly Selected Patient with COPD: A Meta-Analysis

**DOI:** 10.1155/2019/8280358

**Published:** 2019-02-28

**Authors:** Yulin Guo, Feng Cao, Yixuan Ding, Haichen Sun, Shuang Liu, Ang Li, Fei Li

**Affiliations:** Department of General Surgery, Xuanwu Hospital of Capital Medical University, Beijing 100053, China

## Abstract

**Background:**

Laparoscopy has been widely applied in gastrointestinal surgery, with benefits such as less intraoperative blood loss, faster recovery, and shorter length of hospital stay. However, it remains controversial if laparoscopic major gastrointestinal surgery could be conducted for patients with chronic obstructive pulmonary disease (COPD) which was traditionally considered as an important risk factor for postoperative pulmonary complications. The present study was conducted to review and assess the safety and feasibility of laparoscopic major abdominal surgery for patient with COPD.

**Materials and Methods:**

Databases including PubMed, EmBase, Cochrane Library, and Wan-fang were searched for all years up to Jul 1, 2018. Studies comparing perioperative results for COPD patients undergoing major gastrointestinal surgery between laparoscopic and open approaches were enrolled.

**Results:**

Laparoscopic approach was associated with less intraoperative blood loss (MD = −174.03; 95% CI: −232.16 to −115.91, P < 0.00001; P < 0.00001, I^2^=93% for heterogeneity) and shorter length of hospital stay (MD = −3.30; 95% CI: −3.75 to −2.86, P < 0.00001; P = 0.99, I^2^=0% for heterogeneity). As for pulmonary complications, laparoscopic approach was associated with lower overall pulmonary complications rate (OR = 0.58; 95% CI: 0.48 to 0.71, P < 0.00001; P = 0.42, I^2^=0% for heterogeneity) and lower postoperative pneumonia rate (OR = 0.53; 95% CI: 0.41 to 0.67, P < 0.00001; P = 0.57, I^2^=0% for heterogeneity). Moreover, laparoscopic approach was associated with lower wound infection (OR = 0.51; 95% CI: 0.42 to 0.63, P < 0.00001; P = 0.99, I^2^=0% for heterogeneity) and abdominal abscess rates (OR = 0.59; 95% CI: 0.44 to 0.79, P < 0.0004; P = 0.24, I^2^=30% for heterogeneity).

**Conclusions:**

Laparoscopic major gastrointestinal surgery for properly selected COPD patient was safe and feasible, with shorter term benefits.

## 1. Introduction

As with the advances in technology and the improvement in surgical techniques, laparoscopy has been widely applied in the field of gastrointestinal surgery. Compared with open approach (OA), laparoscopic approach (LA) was reported to benefit patients who received major gastrointestinal surgery with less intraoperative blood loss, faster recovery, and shorter length of hospital stay [1–5]. However, it remains controversial if laparoscopic major gastrointestinal surgery could be conducted for patients with chronic obstructive pulmonary disease (COPD).

COPD was identified as an important risk factor for postoperative pulmonary complications (PPCs) following abdominal surgery [6, 7]. Moreover, during the laparoscopic abdominal surgery, the use of carbon dioxide pneumoperitoneum induces reduction in dynamic compliance and functional residual capacity, which increase the risk of hypoxemia, and even respiratory failure for patients with pulmonary morbidities [8, 9]. Therefore, COPD as a diminished pulmonary status was usually considered as a relative contraindication to laparoscopic major abdominal surgery. Recently, several studies reported that laparoscopic major abdominal surgery was well tolerated by patients with COPD, and no difference was found in postoperative pulmonary complications between the laparoscopic and open procedures [10, 11]. Thus, we performed this meta-analysis to assess the safety and feasibility of laparoscopic major abdominal surgery for patients with COPD.

## 2. Methods

### 2.1. Search Strategy for Studies

A systematic search was performed to identify studies that comparing the safety and feasibility of gastrointestinal surgery between laparoscopy and open approaches for patients with COPD. Databases including PubMed, EmBase, Cochrane Library, and Wan-fang database were searched for all years up to Jul 1st 2018. The following search terms and their combinations were used: ((stomach OR gastric) OR gastrectomy), ((((((colon OR colonic) OR rectum) OR rectal) OR colectomy) OR rectectomy) OR proctectomy), (((bowel OR intestine) OR intestinal) OR enterectomy), ((pancreatic OR pancreatoduodenectomy) and pancreatoduodenal), (((laparoscopy OR laparoscopic) OR laparoscope) OR (“minimally invasive”)) and (COPD OR (“chronic obstructive pulmonary disease”)). During the process of study searching, previously published related articles were carefully checked and referenced articles were also searched for assessment.

### 2.2. Inclusion and Exclusion Criteria

The inclusion criteria for studies were as follows: (1) Patients with COPD received resection of gastric, pancreatic, or colorectal lesions. All these surgical procedure should involve resection of bowel, which was defined as complete transection of the lumen at any given point [12, 13]. The diagnosis of COPD was based on clinical symptoms, a certain history of COPD, forced expiratory reserve volume over the first second/forced vital capacity ratio (FEV1/FVC) lower than 0.7 after bronchodilator administration or the value of FEV1 less than 75%. (2) Patients in the experiment group received surgery under the laparoscopic approach, and the control group under the open approach. (3) Outcomes of interest include perioperative mortality and complications. (4) For duplicated data, only better-quality study was included.

The exclusion criteria were as follows: (1) Letters, conferences, unpublished data, and studies of which full data could not be acquired. (2) Noncomparative studies. (3) Resection of adjacent organs other than gallbladder during the surgery. (4) Studies without an English abstract.

Bibliographic citation management software (EndNote X6) was used to manage the retrieved studies. The retrieved studies were assessed by two independent authors through scanning the titles and reviewing the abstracts to identify potential studies. Full texts of the potential studies were further carefully read to assess if they meet with the inclusion criteria. Discussion was conducted to solve any disagreement occurred during the assessment.

### 2.3. Data Extraction and Methodology Quality Assessment

Two authors independently extracted the data. Information extracted includes the followings: first author, year of publication, the number of patients in each group, clinical characteristics, and study type. The outcomes of interest were perioperative results and postoperative complications. Any disagreement occurred during data extraction was resolved by discussion.

Respiratory complications were defined as the occurrence of at least one of the followings: pneumonia, lung atelectasis, pneumothorax, unplanned reintubation, mechanical ventilation for longer than 24 h, respiratory failure, and adult respiratory distress syndrome within 30 days after operation.

The Newcastle-Ottawa Scale (NOS) was employed to assess the quality of cohort study. There are mainly three evaluated items: selection of patients, comparability and controls on the study design, and outcome assessment. Study scored more than six stars was considered as moderate to high quality.

### 2.4. Statistical Analysis and Calculation

Review Manager (Version 5.3) was employed for all the statistical analysis. Mean differences (MDs) and Odds ratios (ORs) were calculated for analyzing continuous and dichotomous data, respectively. For continuous data presented as only median with range, the mean and standard deviation would be estimated as described by Hozo et al. [14]. I2 test was employed to calculate the heterogeneity across studies. When I^2^ value > 50% or p < 0.1, the existence of heterogeneity was indicated and a random effect model would be used. Otherwise, a fixed effect model would be adopted. Sensitivity analysis was employed to detect the strength of the pooled results, for the uncertainty about the data and usage. Sensitivity analysis was carried out through omit one study at a time to assess the effect of any individual study on the overall heterogeneity. The publication bias was assessed by funnel plots. Statistical significance was indicated when the P value < 0.05.

## 3. Results

### 3.1. Search Results and Study Characteristics

**Figure 1:**
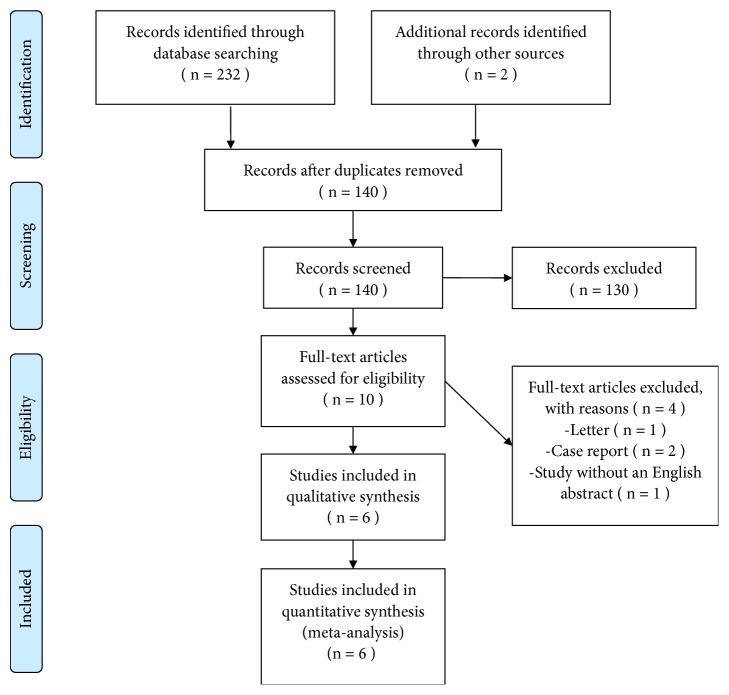
Flow diagram of the study.

A total of 234 relevant citations were obtained based on the search strategy. Among these citations, 94 duplicates were deleted by Endnote software. Then, through scanning titles and abstracts, 130 irrelevant studies, 1 letter, 2 case reports, and 1 study without an English abstract were excluded. Full texts of the remaining 6 studies were reviewed through referring to the inclusion and exclusion criteria. Finally, the 6 cohort studies were included in the present study [10, 11, 15–18].  Figure 1 shows the flow diagram for the inclusion and exclusion process.

The present study enrolled a total of 1356 patients, with 345 patients in the laparoscopic group and 1011 patients in the open group.  Table 1 shows the basic characteristics of the included studies. In the included studies, four studies involved gastrectomy and two involved colectomy. The quality of these included cohort studies were assessed by the NOS. As is shown in Table 1, all these studies scored six or more stars indicating moderate to high quality.

### 3.2. Intraoperative Results

In the present study, perioperative results including operating time and intraoperative blood loss were analyzed. Five studies reported the operating time, with 2846 patients in the laparoscopic group and 2327 in the open group [10, 11, 16–18]. There seemed to be no significant difference in operating time between the two groups (MD = 17.71; 95% CI: −0.88 to 36.29, P = 0.06; P < 0.00001, I^2^=94% for heterogeneity) (Table 2). Intraoperative blood loss was reported in four studies including 776 patients [11, 16–18]. The pooled analysis showed less intraoperative blood loss in the laparoscopic group (MD = −174.03; 95% CI: −232.16 to −115.91, P < 0.00001) with significant heterogeneity (P < 0.00001, I^2^=93%) (Table 2).

### 3.3. Postoperative Results

Postoperative results analyzed in the present study included length of hospital stay and postoperative complications. Three studies reported the length of hospital stay, with 2469 patients in the laparoscopic group and 2144 in the open group [10, 11, 16]. A pooled analysis showed the length of hospital stay in the laparoscopic group was significantly shorter than that in the open group (MD = −3.30; 95% CI: −3.75 to −2.86, P < 0.00001; P = 0.99, I^2^=0% for heterogeneity) (Table 2). The data of overall pulmonary complications were available from all the included studies. The pooled analysis showed the overall pulmonary complications rates were significantly lower in the laparoscopic group compared with that in the open group (OR = 0.58; 95% CI: 0.48 to 0.71, P < 0.00001; P = 0.42, I^2^=0% for heterogeneity) (Table 2).

Two specific pulmonary complications including pneumonia and atelectasis were further analyzed according to the availability of reported data. Pneumonia was reported in five studies, with 2846 patients in the laparoscopic group and 2327 in the open group [10, 11, 16–18]. The pooled analysis showed a significantly lower incidence rate of postoperative pneumonia in the laparoscopic group (OR = 0.53; 95% CI: 0.41 to 0.67, P < 0.00001; P = 0.57, I^2^=0% for heterogeneity) (Table 2). Atelectasis was reported in five studies, involving 893 patients [11, 15–18]. The pooled analysis showed no significant difference considering the incidence rate of postoperative atelectasis between the two groups (OR = 0.46; 95% CI: 0.19 to 1.12, P = 0.09; P = 0.54, I^2^=0% for heterogeneity) (Table 2).

Other postoperative complications were also analyzed, including wound infection, abdominal abscess, postoperative bleeding, anastomotic leakage, pancreatitis fistula, and ileus. Wound infection was reported in five studies, and the laparoscopic approach was associated with lower postoperative incidence rates than the open approach (OR = 0.51; 95% CI: 0.42 to 0.63, P < 0.00001; P = 0.99, I^2^=0% for heterogeneity) (Table 2) [10, 11, 16–18]. Three studies reported abdominal abscess, with 2523 patients in the laparoscopic group and 2206 in the open group [10, 16, 17]. The pooled analysis showed the incidence rate of abdominal abscess was significantly lower in the laparoscopic group compared with that in the open group. (OR = 0.59; 95% CI: 0.44 to 0.79, P < 0.0004; P = 0.24, I^2^=30% for heterogeneity) (Table 2). As for postoperative bleeding, anastomotic leakage, pancreatitis fistula, and ileus, no significant difference was found between the two groups, respectively (All P > 0.05).

### 3.4. Publication Bias

**Figure 2:**
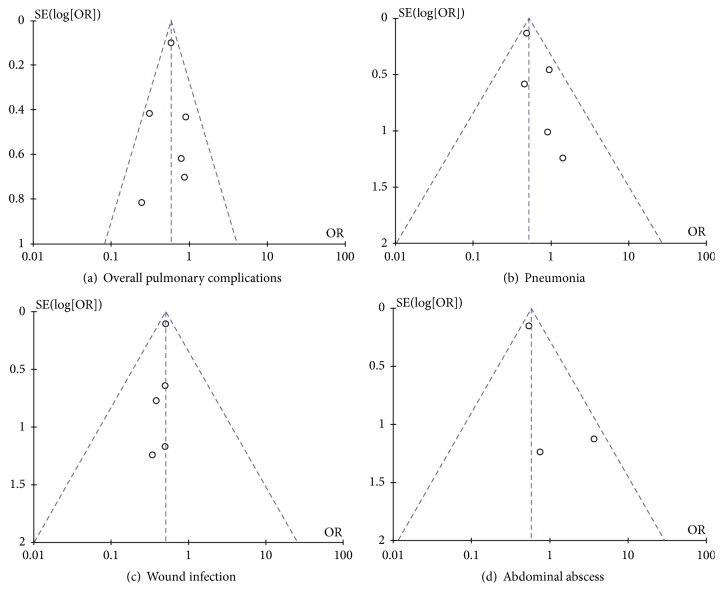
Funnel plot for results from included studies comparing postoperative outcomes between patients with and without COPD. (a) Funnel plot of overall pulmonary complications. (b) Funnel plot of pneumonia. (c) Funnel plot of wound infection. (d) Funnel plot of abdominal abscess.

The funnel plot on postoperative overall pulmonary complications and pneumonia showed none of the included studies lay outside the limits of the 95% CI, indicating there was no serious publication bias (Figures 2(a) and 2(b)). Moreover, there was no serious publication bias for wound infection and abdominal abscess (Figures 2(c) and 2(d)).

### 3.5. Sensitivity Analysis

Because significant heterogeneity was observed in the operating time and intraoperative blood loss, sensitivity analysis was conducted. For operating time, high heterogeneity existed consistently while performing the sensitivity analysis. Moreover, after removal of the Tanigawa et al. study [11], sensitivity analysis found that operating time was significantly longer for the laparoscopic group (MD = 21.61; 95% CI: 0.63 to 42.58, P = 0.04; P < 0.00001, I^2^=95% for heterogeneity). As for intraoperative blood loss, although high heterogeneity existed while performing the sensitivity analysis, the pooled result remained unchanged.

## 4. Discussion

As is known, the laparoscopic approach benefits patients who underwent major gastroenterology resection with less intraoperative blood loss, faster recovery, and shorter length of hospitalization [1–5]. However, for patients with COPD which was reported as an independent risk factor for pulmonary complications after major abdominal surgery, laparoscopic approach may bring with high risk and remains debated [7].

The present study conducted a meta-analysis and found that laparoscopic major gastrointestinal surgery for properly selected COPD patient was feasible and safe, meanwhile conferring the above benefits including less intraoperative blood loss and shorter length of hospital stay brought with laparoscopic approach. Laparoscopic approach for major gastrointestinal surgery was also found associated with less postoperative pulmonary complications and reduced wound infection as well as abdominal abscess.

Recently, along with the development of surgery techniques and perioperative medicine care, laparoscopic surgery was conducted widely, even for patients with COPD. It was reported that laparoscopic cholecystectomy for properly selected patients with COPD was as safe as it for patients without respiratory disease [19]. Moreover, as laparoscopic surgery was associated with less analgesics consumption and postoperative pain, it may benefit patients with better respiratory response [20]. For major gastrointestinal surgery, laparoscopic approach was reported to cause less impaired pulmonary function after surgery compared with the open approach [21, 22]. Study also suggested that the better preserved pulmonary function after the laparoscopic approach for colorectal resection may contribute to reduced pulmonary complications compared with the open approach [23]. Moreover, in the study conducted by Atalay et al., laparoscopic approach for patients with COPD was associated with reduced risk for postoperative pulmonary complications compared with the open approach [24]. The above evidences along with the present study suggest that laparoscopic approach could be as safe as the open approach for properly selected COPD patients who receive the major gastrointestinal surgery.

Apart from the reduced postoperative pulmonary complications brought with the laparoscopic approach for COPD patients found in the present study, laparoscopic major gastrointestinal surgery also benefited these patients with less wound infection. Laparoscopy as a minimally invasive technique has been confirmed could significantly reduce the incidence rate of postoperative surgical site infections [25]. A systematic review was conducted through comparing the surgical site infection rate between laparoscopic and open distal gastrectomy and found that laparoscopic approach was associated with a lower incidence rate of surgical site infection, especially wound infection [26]. A lower incidence of wound infection was also found for patients undergoing laparoscopic resection of colorectal cancer in comparison with those receiving an open approach [27]. As for abdominal abscess, the present study found laparoscopic major gastrointestinal surgery was associated with less abdominal abscess. In the study conducted by Cai et al., abdominal abscess rate was also reported to be reduced for patients undergoing laparoscopic colectomy [28]. However, in the meta-analysis conducted by Xiong et al., the abdominal abscess rate in patients undergoing laparoscopic total gastrectomy was lower than patients undergoing open approach, but the difference was not statistically significant [29]. Moreover, in the more recent systemic reviews and meta-analysis which contained more patients undergoing laparoscopic major gastrointestinal surgeries also reported there were no significant differences in abdominal abscess between the laparoscopic and the open approaches [26, 30, 31]. Thus, debate remains about the surgical approaches regarding abdominal abscess. As for postoperative mortality, two studies stated there were no deaths [15, 16], two studies gave no reports [11, 18], and two studies supported the laparoscopic approach [10, 18]. Because only two studies reported a low rate of mortalities, the pooled analysis was not performed.

Some limitations of the present study should be taken into consideration when interpreting its results. First, as none of the included studies are randomized clinical trials (RCTs), the results of the present study could be affected by the quality of the included studies. Second, variations exist in the protocols, samples and surgical experiences between different clinical centers in each included study, and these might be responsible for the high heterogeneity. Although the random-effects model was adopted when confronting with the heterogeneity, it was impossible to overcome all potential bias. Third, some included studies only provided part of the data about outcomes of interest of the present study. Finally, it is important to bear in mind that for meta-analysis based on published studied, the risk of publication bias always existed, although the funnel plots in the present showed minimal publication bias.

## 5. Conclusion

The present study indicated that laparoscopic major gastrointestinal surgery for the properly selected COPD patient was feasible. Laparoscopic approach not only conferred these patients with less intraoperative blood loss and shorter length of hospital stay, but also benefited them with less postoperative pulmonary complications, reduced wound infection rates, and less abdominal abscess rates. Thus, laparoscopic major gastrointestinal surgery could be safely performed for properly selected COPD patients, with shorter term benefits. Although limitations existed in the present study, to the best of our knowledge this is the first meta-analysis to date focusing on the postoperative outcomes following laparoscopic major gastrointestinal surgery for COPD patient. Better designed RCTs are still needed to confirm our results.

## Figures and Tables

**Table 1:** Perioperative characteristics of patients from the included studies.

First author, year	Number		Age		Gender (male/female)		BMI (kg/m^2^)		ASA class I/II/III/IV		Stage of COPD I/II/III/IV		Smoking		Hypertension		Diabetes		Surgery procedures		NOS Score
	LG	OG	LG	OG	LG	OG	LG	OG	LG	OG	LG	OG	LG	OG	LG	OG	LG	OG	LG	OG	
Tanigawa et al, 2009	61	43	70.0± 8.2	68.9± 9.6	54/7	34/9	22.5± 2.8	21.2± 2.4^*∗*^	9/42/ 10/0	10/28/ 5/0	35/23/ 3/0	26/15/ 1/1	47	29	30	18	10	9	53 partial gastrectomy, 5 subtotal gastrectomy, 3 total gastrectomy	13 partial gastrectomy, 23 subtotal gastrectomy, 7 total gastrectomy^*∗∗*^	8
Huang et al, 2013	74	43	60.00± 10.17	62.51± 10.23	54/20	27/16	NR	NR	NR	NR	36/34/ 4/0	16/24/ 3/0	NR	NR	NR	NR	NR	NR	45 Dixon surgery, 13 right colectomy, 4 left colectomy, 7 Miles surgery, 5 sigmoidectomy	21 Dixon surgery, 10 right colectomy, 2 left colectomy, 3 Miles surgery, 7 sigmoidectomy	8
Inokuchi et al, 2013	115	105	71.2± 7.1	71.2± 8.5^*∗*^	100/15	89/16^*∗*^	23.2± 2.6	22.5± 3.1^*∗*^	NR	NR	57/46/ 11/1	49/42/ 12/2	51	40	42	30	18	21	92 distal gastrectomy, 9 proximal gastrectomy, 14 total gastrectomy	71 distal gastrectomy, 9 proximal gastrectomy, 25 total gastrectomy	7
Yu et al, 2014	262	78	60.9± 7.6	60.4± 7.7	187/75	52/26	20.7± 2.9	21.8± 4.2	145/97/ 20/0	40/31/ 7/0	136/ 108/ 18/0	35/33/ 10/0	135	42	42	20	18	4	137 subtotal gastrectomy, 125 total gastrectomy	46 subtotal gastrectomy, 32 total gastrectomy	9
Hong et al, 2015	44	68	63.1± 7.3	65.9± 7.9	33/11	55/13	NR	NR	5/35/ 4/0	10/50 /8/0	28/16/ 0/0	44/24/ 0/0	23	33	NR	NR	NR	NR	26 subtotal gastrectomy, 18 total gastrectomy	41 subtotal gastrectomy, 27 total gastrectomy	9
Pigazzi et al, 2017	2364	2033	68± 10	69± 10	1167/ 1197	944/ 1089	28±7.5	29±8	0/371/ 1637/ 350	0/221/ 1488/ 318^*∗∗*^	NR	NR	836	770	1648	1406	532	493	1132 partial colectomy, 473 right colectomy, 466 left colectomy	1020 partial colectomy, 433 right colectomy, 401 left colectomy	7

BMI = Body Mass Index; ASA = the American Society of Anesthesiology; COPD = Chronic Obstructive Pulmonary Disease; LG = laparoscopic group; OG = open group; NOS = the Newcastle-Ottawa Scale; NR = not reported.

^*∗*^Significant difference, P < 0.05.

^*∗∗*^Significant difference, P < 0.01.

**Table 2:** Summary of meta-analysis.

Outcome of interest	Statistical method	Number of studies	MD/OR	95% CI	P value	Heterogeneity	
						P	I^2^
Operating time	Random	5	17.71	−0.88, 36.29	0.06	<0.00001^*∗∗*^	94%
Intraoperative blood loss	Random	4	−174.03	−232.16, −115.91	<0.00001^*∗∗*^	<0.00001^*∗∗*^	93%
Length of hospital stay	Fixed	3	−3.30	−3.75, −2.86	<0.00001^*∗∗*^	0.99	0%
Pulmonary complications	Fixed	6	0.58	0.48, 0.71	<0.00001^*∗∗*^	0.42	0%
Pneumonia	Fixed	5	0.53	0.41, 0.67	<0.00001^*∗∗*^	0.57	0%
Atelectasis	Fixed	5	0.46	0.19, 1.12	0.09	0.54	0%
Wound infection	Fixed	5	0.51	0.42, 0.63	<0.00001^*∗∗*^	0.99	0%
Abdominal abscess	Fixed	3	0.59	0.44, 0.79	0.0004^*∗∗*^	0.24	30%
Postoperative bleeding	Fixed	3	0.40	0.08, 2.04	0.27	0.53	0%
Anastomotic leakage	Fixed	4	0.94	0.40, 2.25	0.90	0.93	0%
Pancreatitis fistula	Fixed	3	0.82	0.31, 2.20	0.70	0.27	24%
Ileus	Fixed	4	0.61	0.28, 1.31	0.20	0.76	0%

MD = mean difference, OR = odds ratio, and CI = confidence interval.

^*∗*^Significant difference, P < 0.05.

^*∗∗*^Significant difference, P < 0.01.

## Data Availability

The data supporting this meta-analysis are from previously reported studies and datasets, which have been cited. The processed data are available from the corresponding author upon reasonable request.

## Abbreviations

COPD: Chronic Obstructive Pulmonary Disease
NOS: Newcastle-Ottawa Scale
OR: Odds ratios
MD: Mean difference
CIs: Confidence intervals
OA: Open approach
LA: Laparoscopic approach
PPCs: Pulmonary complications
FEV1: The forced expiratory volume in 1 second (FEV1)
FVC: Forced vital capacity, randomized clinical trials (RCTs).
